# Translation into French of: “Changes to publication requirements made at the XVIII International Botanical Congress in Melbourne – what does e-publication mean for you?”. Translated by Christian Feuillet and Valéry Malécot Changements des conditions requises pour la publication faits au XVIII ^e^ Congrès International de Botanique à Melbourne – qu’est-ce que la publication électronique représente pour vous?

**DOI:** 10.3897/phytokeys.7.2195

**Published:** 2011-11-29

**Authors:** Sandra Knapp, John McNeill, Nicholas J. Turland

**Affiliations:** 1 Department of Botany, The Natural History Museum, Cromwell Road, London SW7 5BD, UK; 2 Royal Botanic Garden, Edinburgh, 20A Inverleith Row, Edinburgh EH3 5LR, UK; 3 Missouri Botanical Garden, PO Box 299, St Louis, MO 63166-0299, USA

## Abstract

Les changements au *Code* *International de Nomenclature Botanique* sont décidés tous les 6 ans aux Sections de Nomenclature associées aux Congrès Internationaux de Botanique (CIB). Le XVIII^e^ CIB se tenait à Melbourne, Australie; la Section de Nomenclature s’est réunie les 18-22 juillet 2011 et ses décisions ont été acceptées par le Congrès en session plénière le 30 juillet. Suite à cette réunion, plusieurs modifications importantes ont été apportées au *Code* et vont affecter la publication de nouveaux noms. Deux de ces changements prendront effet le 1^er^ janvier 2012, quelques mois avant que le *Code de Melbourne* soit publié. Les documents électroniques publiés en ligne en ‘Portable Document Format’ (PDF) avec un ‘International Standard Serial Number’ (ISSN) ou un ‘International Standard Book Number’ (ISBN) constitueront une publication effective, et l’exigence d’une description ou d’une diagnose en latin pour les noms des nouveaux taxa sera changée en l’exigence d’une description ou d’une diagnose en latin ou en anglais. De plus, à partir du 1^er^ janvier 2013, les noms nouveaux des organismes traités comme champignons devront, pour que la publication soit valide, inclure dans le protologue (tous ce qui est associé au nom au moment de la publication valide) la citation d’un identifiant (‘identifier’) fourni par un dépôt reconnu (tel MycoBank). Une ébauche des nouveaux articles concernant la publication électronique est fournie et des conseils de bon usage sont esquissés.

Pour encourager la diffusion des changements adoptés au Code International de Nomenclature pour les algues, les champignons et les plantes, cet article sera publié dans *BMC Evolutionary Biology, Botanical Journal of the Linnean Society, Brittonia, Cladistics, MycoKeys, Mycotaxon, New Phytologist, North American Fungi, Novon, Opuscula Philolichenum, PhytoKeys, Phytoneuron, Phytotaxa, Plant Diversity and Resources, Systematic Botany* et *Taxon*.

## Introduction

En juillet 2011, durant le XVIII^e^ Congrès International de Botanique à Melbourne, Australie, deux modifications importantes ont été apportées au *Code International de Nomenclature Botanique* (maintenant appelé *Code International de Nomenclature pour les Algues, les Champignons et les Plantes*) qui prendront effet au 1^er^ janvier 2012. Ces changements affecteront toutes les personnes qui publieront des noms régis par ce *Code*. Comme le *Code de Melbourne* ne sera pas publié avant mi-2012, nous avons pensé qu’il serait utile de donner les grandes lignes de ces modifications, en particulier celles concernant la publication effective sur supports électroniques (dans les Articles 29, 30 et 31). Pour un rapport concis sur tous les changements au *Code* acceptés à Melbourne, voyez McNeill et al. (2011).

Une ébauche des Articles, Notes et Recommandations traitant de publication effective est fournie pour aider les rédacteurs et les éditeurs à établir les meilleures pratiques pour mettre en œuvre cette partie du *Code*. Nous précisons aussi ici ce que ces modifications *ne* signifient *pas*, pour guider ceux qui souhaitent publier des noms nouveaux et des typifications sur supports électroniques. Nous conseillons aux lecteurs de consulter le rapport du Comité Spécial sur la Publication Électronique accompagnant les changements proposés avant le Congrès (Chapman et al. 2010), où les raisons des changements maintenant acceptés dans le *Code* sont présentées.

## Canevas des Articles 29, 30 et 31, et des Recommandations 29A, 30A et 31A modifiés

Ici nous reproduisons le texte de tous les Articles, Notes et Recommandations pertinents (sauf les exemples), avec les changements surlignés en **gras**. La rédaction est ici provisoire, dans l’attente de la réunion du Comité Éditorial en décembre 2011 pour finaliser la version imprimée du *Code de Melbourne*.

### Article 29

*29.1.* Unepublication n’est effective, aux termes de ce *Code*, que par la distribution de documents imprimés (par vente, échange ou don) au public en général ou, au minimum, à des institutions botaniques dont les bibliothèques sont accessibles aux botanistes en général. **Une publication est aussi effective par distribution par voie électronique de matériel en ‘Portable Document Format’ (PDF; voir aussi l’Art. 29.3 et la Rec. 29A.1) dans une publication en ligne avec un ‘International Standard Serial Number’ (ISSN) ou un ‘International Standard Book Number’ (ISBN).** Une publication n’est pas effective par la communication de noms nouveaux dans une réunion publique, par l’apposition de noms dans des collections ou des jardins ouverts au public, par l’édition de microfilms reproduisant des textes manuscrits ou dactylographiés ou tout autre matériel non publié, ou par une distribution électronique **autre que celles décrites ci-dessus.**

***29.2.* Dans le cadre decet Article, ‘en ligne’ est défini comme accessible électroniquement sur le ‘World Wide Web’.**

***29.3.* Si le ‘Portable Document Format’ (PDF) devenait obsolète, un format standard international successeur communiqué par le Comité Général (voir Div. III) est acceptable.**

***29.4.* Le contenu d’une publication électronique particulière ne doit pas être altéré après sa première parution. Aucune de ces altérations n’est elle-même effectivement publiée. Des corrections ou des révisions doivent paraître séparément pour être effectivement publiées.**

### Recommandation 29A

[La Recommandation existante est remplacée par ce qui suit:]

***29A.1.* Une publication électronique au ‘Portable Document Format’ (PDF) devrait se conformer au standard d’archivage PDF/A (ISO 19005).**

***29A.2.* Les auteurs devraient, en préférence, publier dans des publications qui sont archivées, autant que possible en conformité avec les critères suivants (voir aussi la Rec. 29A.1) :**

**(*a*) Le matériel devrait être déposé dans plusieurs dépôts électroniques en ligne réputés, c’est dire un dépôt certifié ISO ;**

**(*b*) Les dépôts électroniques devraient être dans plus d’une région géographique du monde et de préférence sur des continents différents ;**

**(*c*) Le placement de copies imprimées dans des bibliothèques dans plus d’une région géographique du monde et de préférence sur des continents différents est également recommandé.**

### Article 30

***30.1.* La publication par diffusion de matériel électronique ne constitue pas une publication effective avant le 1^er^ janvier 2012.**

***30.2.* Les publications électroniques ne sont pas effectives si il y a dans, ou associée à, la publication une preuve que la publication est seulement une version préliminaire qui a été, ou doit être, remplacée par une version que l’éditeur considère comme finale, auquel cas seule cette version finale est effectivement publiée.**

*30.3*. La publication, avant le 1^er^ janvier 1953, par autographie indélébile est effective. Une autographie indélébile publiée à une date ultérieure n’est pas effectivement publiée.

*30.4.* Dans le cadre de cet Article, une autographie indélébile est un document manuscrit reproduit par un processus mécanique ou graphique (tel que la lithographie, l’offset ou la gravure sur métal).

*30.5.* La publication au ou à partir du 1^er^ janvier 1953, dans les catalogues commerciaux ou les journaux d’information non scientifique, et au ou à partir du 1^er^ janvier 1973, dans les listes d’échange de graines, ne constitue pas une publication effective.

*30.6.* La distribution, au ou à partir du 1^er^ janvier 1953, de document imprimé accompagnant des exsiccata ne constitue pas une publication effective.

*Note 1.* Si le document imprimé est également distribué indépendamment des exsiccata, il est effectivement publié.

*30.7.* La publication, au ou à partir du 1^er^ janvier 1953, d’un travail indépendant isolé dit être une thèse soumise à une université ou un autre établissement d’enseignement dans le but d’obtenir un diplôme n’est pas effectivement publié à moins qu’il contienne une déclaration explicite (faisant référence aux dispositions du *Code* pour une publication effective) ou une autre preuve interne qu’il est considéré comme une publication effective par son auteur ou éditeur.

*Note 2.* La présence d’un ‘International Standard Book Number’ (ISBN) ou la mention d’un nom d’imprimeur, d’éditeur ou de distributeur dans la version imprimée originale est considérée comme une évidence interne que ce travail était destiné à être effectivement publié.

### Recommandation 30A

***30A.1.* Les versions préliminaire ou finale d’une même publication électronique devraient être clairement indiquées comme telles au moment de leur première parution.**

*30A.2.* Il est vivement recommandé aux auteurs d’éviter de publier de nouveaux noms et des descriptions ou diagnoses de nouveaux taxons dans un document imprimé éphémère de n’importe quel type, notamment dans un document imprimé qui est multiplié en nombre limité et incertain, dont la persistance du texte peut être limitée, dont la publication effective du point de vue du nombre d’exemplaires n’est pas évidente, ou qui n’ont guère de chance d’atteindre le public. Les auteurs devraient aussi éviter de publier des noms nouveaux et des descriptions ou diagnoses dans des périodiques populaires, dans des périodiques de documentation (« abstracting journals ») ou sur des feuilles d’errata.

*30A.3.* Pour favoriser la disponibilité dans le temps et l’espace, les auteurs publiant des nouveautés nomenclaturales devraient donner la préférence aux périodiques qui publient régulièrement des articles taxinomiques. **Autrement, une copie d’une publication (qu’elle soit publiée sous forme imprimée ou électronique) devrait être envoyée au(x) centre(s) d’indexation approprié(s) pour le groupe taxonomique, et les publications qui existent seulement sous forme imprimée** devraient être déposées dans au moins dix - mais de préférence plus - bibliothèques botaniques ou des bibliothèques généralement accessibles à travers le monde.

*30A.4.* Les auteurs et les rédacteurs sont encouragés à mentionner les nouveautés nomenclaturales dans le sommaire ou le résumé, ou à les lister dans un index dans la publication.

### Article 31

*31.1.* La date de publication effective est la date à laquelle le document imprimé **ou électronique** devient disponible ainsi que définit dans les Art. 29 et 30. En l’absence de preuve établissant une autre date, celle qui figure sur le matériel imprimé **ou électronique** lui-même doit être acceptée comme correcte.

 [La Note 1 existante est remplacée par ce qui suit :]

***31.2.* Quand une publication parait en parallèle en versions électronique et imprimée, celles-ci doivent être traitées comme publiées effectivement à la même date, à moins que les dates des versions soient différentes au sens de l’Art. 31.1.**

*31.3.* Lorsque les tirés-à-part de périodiques ou d’autres ouvrages mis en vente sont distribués à l’avance, la date sur le tirés-à-part est acceptée comme la date de publication effective, à moins qu’il y ait une preuve qu’elle est erronée.

### Recommandation 31A

*31A.1.* La date à laquelle l’éditeur ou son agent remet le document imprimé à l’un des transporteurs usuels pour la distribution au public devrait être acceptée comme sa date de publication effective.

## Bon usage

Les auteurs de noms nouveaux, rédacteurs et éditeurs auraient intérêt à s’assurer que les publications comprenant des noms nouveaux sont en accord avec le *Code de Melbourne*, pour que ces noms soient effectivement publiés. Nous suggérons que ceux qui publient dans des journaux ou des séries monographiques et des livres qui ont des éditions en ligne communiquent avec les éditeurs pour qu’un bon usage puisse être établi dans la communauté aussi vite que possible. De nombreux éditeurs ont été attentifs depuis quelques années aux problèmes liés à la publication électronique (‘e-publication’) des nouveautés taxonomiques (cf. Knapp and Wright 2010; guidelines in PLoS One [http://www.plosone.org/static/policies.action#taxon]) et un intérêt considérable pour rendre fonctionnelles les modifications de ce nouveau *Code* a été apparent.

Certaines pratiques, dont nous pensons qu’elles aideront les étapes initiales de l’e-publication de nouveautés faits en accord avec le *Code de Melbourne*, sont les suivantes :

•	Avoir dans chaque article la date de publication en position évidente (comme c’est le cas de nombreux journaux, par exemple *New Phytologist* ou *Nature*).

•	Si une version mise en ligne en avance paraît, et qu’elle n’est pas la même que la version finale (et donc qu’elle n’est pas le lieu d’une publication effective), estampillez de manière évidente chaque article avec la mention de cet état de fait (par exemple *American Journal of Botany*).

•	Afficher de manière évidente les ISSN ou ISBN de la publication sur chaque article aidera les indexeurs à établir que la publication est effective.

•	Publier dans des journaux (ou des séries monographiques) qui participent au système CLOCKSS (cf. Knapp and Wright 2010 pour une description) ou un autre système international d’archivage et de préservation assurera un archivage à long terme.

•	Les auteurs de nom nouveau sur support électronique devraient alerter les centres d’indexation appropriés comme recommandé par la Rec. 30A.3 - cela aidera les indexeurs qui pourraient autrement ne pas être au courant de noms publiés électro niquement.

### Ce que ces changements ne signifient pas

Bien que les nouveaux Articles et Recommandations utilisent les termes PDF et PDF/A, cela ne veut pas dire que les publications doivent paraître *seulement* dans ce format pour être effectivement publiées. Par exemple, certains journaux en ligne font paraître des articles au format Hypertext Markup Language (HTML) avec une version parallèle PDF. Dans ce cas, la version PDF sera effectivement publiée. La mention disant que le Comité Général pour la Nomenclature Botanique communiquera les nouveaux standards internationaux acceptables, si le PDF devenait obsolète, signifie que les auteurs de nouveaux noms et la communauté des utilisateurs du *Code* pourront rester informé des développements dans cette discipline et que le *Code* sera protégé de l’obsolescence.

L’utilisation des supports suivants pour la publication électronique ne résultera pas en une publication effective des noms nouveaux d’après le *Code de Melbourne* :

•	La publication sur des sites web ou dans des documents éphémères disponibles sur Internet (il y a des critères stricts pour attribuer des ISSN [http://www.issn.org]).

•	La publication dans des journaux sans un ISSN ou e-ISSN enregistré.

•	La publication dans des livres sans un ISBN or e-ISBN enregistré.

La Recommandation adoptée de conseiller le dépôt d’une copie imprimée de chaque e-publication dans une bibliothèque suggère une action aux botanistes, mais elle n’établit pas une pratique standard ou un protocole à suivre pour les bibliothécaires. Les bibliothécaires sont eux-mêmes dans une phase de transition complexe entre des modalités de publication (Johnson and Luther 2007), et les botanistes pourraient trouver les bibliothécaires réticents ou incapables d’accepter des articles séparés imprimés en accessions individuelles si le volume en est important.

### Deux autres modifications importantes dans le Code concernant la publication des noms

Le second changement au *Code* adopté à Melbourne pour prendre effet à partir du 1^er^ janvier 2012 est que la description ou la diagnose requise pour une publication valide du nom d’un nouveau taxon pour tous les organismes régis par le *Code* peut être soit en anglais soit en latin. C’est la règle actuelle pour les noms de fossiles végétaux, mais tous les nouveaux taxa non-fossiles requéraient une description ou une diagnose en latin (champignons et plantes depuis le 1^er^ janvier 1935; algues [y compris les cyanobactéries, si traitées sous le *Code*] depuis le 1^er^ janvier 1958). Cela n’a aucune influence sur la forme des noms scientifiques, qui continuent d’être latins ou réputés latins. Les exigences de chaque journal vis à vis du latin et/ou de l’anglais seront, bien sûr, déterminées par le rédacteur de chaque journal.

Un troisième changement au *Code* adopté à Melbourne au sujet de la publication des noms, qui ne prendra effet que le 1^er^ janvier 2013 (pas le 1^er^ janvier 2012 comme déclaré par Miller et al. 2011), énonce que tous les noms d’organismes traités comme des champignons doivent, comme condition supplémentaire pour une publication valide, inclure dans le protologue (tous ce qui est associé au nom au moment de la publication valide) la citation d’un identifiant fourni par un dépôt reconnu (tel que MycoBank [http://www.mycobank.org/]). Cela sera rendu public par ailleurs.

L’exigence d’un identifiant unique pour les nouveaux noms de champignon à partir du 1^er^ janvier 2013 *ne* s’applique *pas* aux plantes ou aux algues; il n’y a pas besoin pour les auteurs de nouveaux noms dans ces groupes de demander des ‘Life Science Identifiers’ (LSIDs) - ou d’autres identifiants – aux centres d’indexation.

## Remerciements

SK est appuyé par le programme de la NSF Planetary Biodiversity Inventory (DEB-0316614, ‘PBI Solanum - a worldwide treatment’). La participation de JMcN et de NJT à la Section de Nomenclature du XVIII^e^ CIB à Melbourne a été supportée en partie par l’International Association for Plant Taxonomy (IAPT). Nous remercions Katherine Challis (Kew) pour ses commentaires utiles.

Pour encourager la dissémination des modifications apportées au Code International de Nomenclature pour les algues, les champignons et les plantes, cet article sera publié dans *BMC Evolutionary Biology, Botanical Journal of the Linnean Society, Brittonia, Cladistics, MycoKeys, Mycotaxon, New Phytologist, North American Fungi, Novon, Opuscula Philolichenum, PhytoKeys, Phytoneuron, Phytotaxa, Plant Diversity and Resources, Systematic Botany* et *Taxon*.

## Référence supplémentaire pour la traduction en français:

Malécot V, Soca R (2002) Code International de Nomenclature Botanique de St Louis, Adopté par le Seizième Congrès International de Botanique, St Louis, Missouri, Juillet–Août 1999. (traduction française) http://www.tela-botanica.org/page:code_botanique_st_louis?wiki=CinbTraduction (consulté le 28 septembre 2011)

[À notre connaissance, il n’y a pas de traduction française du Code de Vienne 2006 ; les traductions proposées ci-dessus pour des portions non modifiées des articles du Code de Vienne sont pour la plupart inédites et respectent à la lettre le texte de 2006 et non pas celui de 2000.]
